# Response of plant diversity and soil physicochemical properties to different gap sizes in a *Pinus massoniana* plantation

**DOI:** 10.7717/peerj.12222

**Published:** 2021-09-21

**Authors:** Qian Lyu, Jiangli Liu, Junjie Liu, Yan Luo, Luman Chen, Gang Chen, Kuangji Zhao, Yuqin Chen, Chuan Fan, Xianwei Li

**Affiliations:** 1 College of Forestry, Sichuan Agricultural University, Chengdu, China; 2 Key Laboratory of National Forestry and Prairie Bureau on Forest Resources Conservation and Ecological Security in the Upper Reaches of Yangtze River (Sichuan Agricultural University), Chengdu, China

**Keywords:** Soil properties, Undergrowth plant diversity, Forest gap size, Forest plantation, Close-to-nature management

## Abstract

As one means of close-to-nature management, forest gaps have an important impact on the ecological service function of plantations. To improve the current situation of *P. massoniana* plantations, three different sizes of forest gaps (large gaps, medium gaps and small gaps) were established to observe whether gap setting can improve the soil fertility and plant diversity of forest plantations. The results showed that compared with the control, the soil organic matter content of different soil layers increased significantly in the medium forest gap and large forest gap. The content of soil organic matter in the surface layer of the middle gap had the largest increase (80.64%). Compared with the control, the content of soil-available potassium between different soil layers decreased significantly by 15.93% to 25.80%. The soil hydrolysable nitrogen reached its maximum under the medium gap. Soil moisture showed significant changes among different gap treatments, different soil layers and their interaction, decreasing significantly in large gaps and small gaps but increasing significantly in medium gaps. The soil bulk density decreased significantly compared with the control, and the surface soil reached the minimum in the medium gap. There were different plant species in forest gaps of different sizes, and shrub layer plants were more sensitive to gap size differences than herb layer plants. The plant diversity indices of the shrub layer increased significantly and showed a maximum under the medium gap. The plant diversity of the herb layer showed the opposite trend, and the Shannon-Wiener index, Simpson index and Pielou index were significantly lower than those of the control. RDA showed that different gap treatments had significant effects on the distribution of plants under the forest. Soil available potassium, soil moisture and soil bulk density affected the distribution and diversity of plants under the forest, serving as the limiting factors of plant growth. In forest management, if we strictly consider the improvement of plant diversity and soil physicochemical properties, these results suggest that a medium gap should be established in a plantation for natural restoration.

## Introduction

Declines in forest quantity and quality can contribute to a series of global environmental problems, such as global warming, loss of biodiversity, soil erosion and site quality decline (Feng et al., 2016; Li & Chen, 2020b). China’s forestry development has moved from quantitative growth to a new stage with equal emphasis on both quantity and quality, and the continuous expansion of plantations has already raised concerns about threats to biodiversity from both the public and scientists (Badalamenti et al., 2018; Zhou et al., 2020). In China, the total area of plantation forests is approximately 6. 9 × 10^7^ ha according to the eighth National Forest Resources Inventory survey; this amount accounts for approximately a quarter of the world’s plantation area (Chen et al., 2015; Mo et al., 2020). *Pinus massoniana* Lamb., as an important fast-growing economic timber species in southern China, is widely distributed in the northern subtropics, mid-subtropics, southern subtropics and northern tropics of China and occupies an extremely important position in forestry production and forests (Li et al., 2019; Huang et al., 2020). *P. massoniana* is a pioneer tree species in community succession in the low mountain and hilly region of eastern Sichuan because of its strong adaptability and resistance to drought and barren land; however, the large, single-species forests of *P. massoniana* combined with improper forest management measures, have led to differentiation of the internal ecological microenvironment. Many ecological health concerns, such as the decrease in aboveground and underground biodiversity and the weakening of carbon sequestration functions, have seriously restricted the sustainable development of local forestry (Li et al., 2020d). In close-to-nature forestry, obtaining different sizes of forest gaps by cutting stands and implementing small- and medium-scale man-made interference is an important means for promoting plantation regeneration, close-to-nature cultivation and ecological service function improvement (Angers et al., 2005; Zeibig, Diaci & Wagner, 2005). Forest gaps are an effective way to solve a series of ecological health concerns caused by plantations. They can promote stand regeneration, increase biodiversity and improve stand structure, which is an important means for close-to-nature management of forest plantations to improve stand quality (Emborg, 1998; Hubbell et al., 1999; Lu et al., 2018).

The formation of forest gaps changes the forest structure and microenvironmental conditions (light, temperature, relative humidity, etc.), so the gap affects heat transfer at different positions in the gap through changes in thermal characteristics (Gutiérrez del Arroyo & Silver, 2018; Tena et al., 2020). It can further affect the physical and chemical properties of soil, the decomposition of organic matter and the activity of soil microorganisms. Finally, it can also lead to changes in ecological processes in and around the forest gap, thus affecting the renewal and distribution of species (Perreault et al., 2020). However, the effects of forest gaps on soil properties and forest biodiversity are not clear. Understory vegetation is crucial and necessary for stability and biodiversity in plantation forests (Gracia et al., 2007; Ahmad et al., 2019; Mo et al., 2020). Gap formation also contributes to the dynamic growth of woody plants. Large-area gaps form mosaic patches with different sizes of tree species, structures and ages (Runkle, 1981; Nygaard, Strand & Stuanes, 2018; Keram et al., 2019). Forest soil, an essential part of the ecosystem in which nutrients are recycled and many organisms live, is also influenced by forest canopy gaps (Yang et al., 2017). Changes in the forest environment caused by gaps can exert significant impacts on soil material cycling and nutrient turnover (Han et al., 2020). He et al. (2013) found that in the *Castanopsis kawakamii* Nature Forest, the disturbance of forest gaps could improve the soil physical and chemical properties and increase the population species richness, which would provide an ecological basis for species coexistence in the *Castanopsis kawakamii* natural forest. In a temperate hardwood forest, canopy gaps largely determine light transmission to lower canopy strata, thereby controlling the turnover of tree individuals in the stand and increasing the species richness and abundance of woody taxa (Feldmann et al., 2018; Sabo et al., 2019). Gap size is an important contributing factor for defining local plant community colonization. The changes in soil physical and chemical properties are different under different forest gaps, and there is no consistency among different forest ecosystems (Thiel & Perakis, 2009; Kathke & Bruelheide, 2010; Wang & Liu, 2011; Mallik, Kreutzweiser & Spalvieri, 2014; Bossard et al., 2015). It has been proven that due to the favorable light and soil temperature in medium to large gaps, the density and diversity of the woody plant community in these gaps is higher than that in small gaps (Mallik, Kreutzweiser & Spalvieri, 2014). In addition, the introduction of gaps has also proven that the effect of gap size on the soil microenvironment provides valuable information for evaluating the response of soil phosphorus and other nutrients to forest management (Hu et al., 2016). However, at the same time, there may be areas with poor fertility in larger forest gaps, which will reduce the availability of soil nutrients in a *Cunninghamia lanceolata* stand (Xu, Xue & Su, 2016).

*P. massoniana*, as a plantation species with important economic and ecological value in China, accounts for more than 7.7% of the total forest area in the country (Zeng et al., 2018; He et al., 2020). Due to the high stand density, single tree species composition and lack of long-term management, the biodiversity in the forest is low and the stand ecosystem is fragile. How to optimize its structure, improve ecological service function and accurately enhance forest quality has become an urgent problem to be solved in *P. massoniana* plantation. Therefore, in this study, through artificial cutting of three kinds of forest gaps of different sizes, and with *P. massoniana* plantation without open gaps as control, the soil physical and chemical properties, plant diversity and the interaction among them were analyzed in a *P. massoniana* plantation underwent close-to-nature management by artificially create forest gaps at different sizes. The purpose of this study is to provide scientific reference for promoting sustainable management of plantation in similar conditions. We hypothesized that the physical properties of the soil will be reduced as the forest gap can cause a change in light heterogeneity in the forest. We further hypothesized that forest gaps will increase the richness and diversity of plants in the shrub and herb layers and that, under the comprehensive action of gap size and soil physical and chemical properties, the composition of community vegetation will change.

## Material and Methods

### Study area

**Figure 1:**
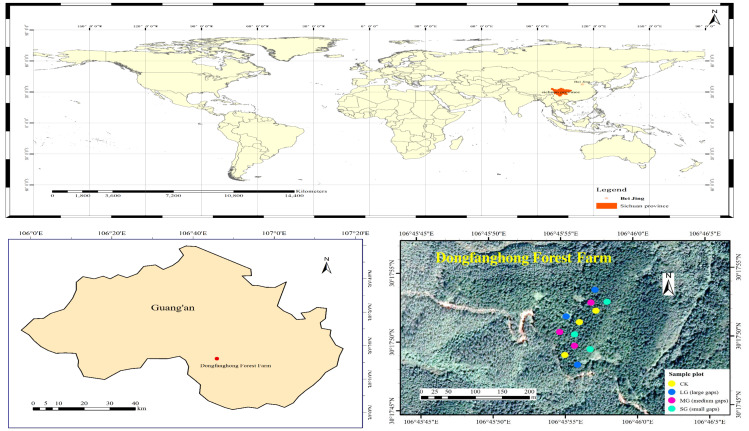
Location of the study area and distribution of study plots.

The study area is located in Dongfanghong Forest Farm, Huaying City, Sichuan Province, in the low mountain area of eastern Sichuan (106°45′53.27E– 106°45′55.87E, 30°17′48.45N–30°17′55.09N) (Fig. 1). Elevations range from 544.1 to 574.7 m. Climatic characteristics of the region include a mild climate, abundant rainfall, uneven rainfall and large temperature differences. The multiyear average temperature in Huaying is 17.2 °C. The area has abundant rainfall, with a maximum annual average of 1441.7 mm, a minimum annual average of 854.9 mm, and a multiyear average of 1087.84 mm. The soil type is typically a low-fertility yellow soil (Nitisol) in the study area (Li et al., 2020d). The shrub layer in the forest gaps of different areas consists mainly of *Cinnamomum camphora* (L.) Presl, *Quercus serrata* Murray and *Mallotus barbatus* (Wall.) Muell. Arg. The herb layer is mainly composed of ferns.

### Experimental design and data collection

The field plot design was the *P. massoniana* plantation in the 1870S, which had similar site conditions, the same forest age and the same management history in Dongfanghong Forest Farm. In early August 2017, we used laser distance meter (LDM-80H) to determine forest gap sizes, then marked the *P. massoniana* that needed to be cut with red paint. The experiment was a complete randomized design, and nine artificial gaps were created with gap sizes ranging from 50 m^2^ to 667 m^2^ (Fig. 1). Furthermore, these gaps were divided into three levels, *i.e.,* small gaps (SG): 50–100 m^2^ (*n* = 3), medium gaps (MG): 100–200 m^2^ (*n* = 3) and large gaps (LG): 400–667 m^2^ (*n* = 3), surrounded by closed canopy transition zones and a 5 m buffer. All gaps were approximately circular and located in similar topography, with aspects southwest-facing, slopes ranging from 7 to 17° . The mean height of gap border trees was 15 m, with a mean DBH of 22 cm. A designated sample plot without any management was also established as the control plot with three replicates. We set each of the control plots to be 20 m ×20 m. The trunks and branches of *P. massoniana* cut down in the forest gap were all removed from the gap, and plant regeneration in the gap mainly depended on seeds and the soil seed bank.

Vegetation surveys were conducted in October 2019. Five 3 m ×3 m squares were mechanically arranged as shrub squares in the four corners and the center of sample squares within different gap areas of each *P. massoniana* plantation, and then ten 1 m ×1 m squares were set up in each of the shrub squares as herb squares. We counted and recorded the species name, the number of plants and the branch diameter and height of each plant in the shrub quadrats. The average height and branch diameter of each shrub in the plot were measured using a tape line. In the herb layer, we counted and recorded the species name, overall coverage, average coverage and average height of each species occurring in the herb quadrats. The indicator species are that the important values of shrub layer plants are more than 5% and that of herb layer plants are more than 10% (Wang et al., 2019b).

According to the data obtained from the sample plot survey, we calculated the importance value of the plants appearing in the sample plot and then calculated the diversity index. The important value is an important index in calculating and evaluating species diversity, and the comprehensive value is used to express the relative importance of plant species in the community. The IV value was used to calculate species diversity in each plot (Zhang et al., 2011). We used four *α*-diversity indices: Richness (*R*), the Shannon-Wiener index (*H*), Simpson index (*D*) and Pielou index (*J*) (Zhou et al., 2016), which were calculated as follows:

Important value (%): IV = (Relative density + Relative frequency + Relative coverage) /3 (1) (Ming et al., 2020)

(2) \begin{eqnarray*}R=S\end{eqnarray*}

(3) \begin{eqnarray*}\text{Simpson index}:D=1-\sum _{\mathrm{i=1}}^{\mathrm{s}}{\mathrm{P}}_{\mathrm{ i}}^{2}\end{eqnarray*}

(4) \begin{eqnarray*}\text{Shannon-Wiener index}:H=-\sum _{\mathrm{i}=1}^{\mathrm{s}}{\mathrm{P}}_{\mathrm{ i}}\ln \nolimits {P}_{i}\end{eqnarray*}

(5) \begin{eqnarray*}\text{Pielou index}:J=H/\ln \nolimits \mathrm{S}\end{eqnarray*}

Where S is the total number of species in the plot; i is species i; and P_i_ is the relative importance value of species i.

A soil drill (five cm in diameter) was used in each crop tree management stand to randomly arrange five sampling points in an “S” shape to collect soil samples. Soil samples at depths of 0–10 cm and 10–20 cm were collected separately at each sampling point and mixed evenly. Approximately 1.5 kg of each sample was selected according to the quartet method, packed into plastic bags and taken back to the laboratory within 24 h. Fine roots, fine gravel and litter were manually separated from the soil. Then, the soil samples were air-dried and passed through two mm and 0.149 mm sieves. A subsample was dried at 105 °C for 48 h to constant weight to measure the soil gravimetric moisture content and soil bulk density (Luo et al., 2018). The organic matter content of the soil was determined by hydration with the potassium dichromate oxidation-colorimetric method (Yang et al., 2020). A 1:2.5 water and soil mixture was whisked together for 10 min with a glass rod, left to stand for 1 h, and then measured for pH with an electronic meter (Li et al., 2020d). Soil hydrolysable nitrogen was measured by the alkali-hydrolysed diffusion absorption method (Chen et al., 2010). Soil hydrolysable nitrogen was measured by the alkali-hydrolyzed diffusion absorption method (Rich, 1958). Soil available phosphorus was measured by extracting subsamples with 0.03 M NH_4_F –0.025 M HCl. (Mariotte et al., 2020).

### Statistical analyses

Single factor analysis of variance (one-way ANOVA) and multiple comparisons (LSD) were used to test the soil index and plant diversity index. Two-way ANOVA was used to test whether there was a significant interaction among the different forest gaps, different soil layers and soil physicochemical properties. Significant differences were detected at *p* < 0.05. Pearson correlation analysis was carried out between the plant diversity index and the soil physicochemical properties. Redundancy analysis was carried out by using CANOCO5.0 software to compare the correlation between the plant composition of the shrub and herb layers and environmental factors in different forest gaps. All data were processed by Excel software, Origin Pro 8.0 was used to create the figures, and statistical analysis was performed using SPSS 20.0.

## Results

### Soil physicochemical properties

According to Table 1, soil pH was not significantly different in the different forest gaps, and it was acidic and fluctuated in a small range, but there was a significant difference between the two soil layers (Table 2). Compared with the control, the content of soil organic matter increased significantly under the middle gap and small gap, and the content of soil organic matter reached the maximum in the 0–10 cm soil layer of the medium gap, which increased by 80.64%. There was no significant difference in soil-available phosphorus content between the different soil layers of each treatment. The content of soil hydrolyzed nitrogen was significantly affected by different forest gap treatments and interactive treatments between different forest gaps and different soil layers (Table 2). Compared with the control, the content of soil hydrolyzed nitrogen significantly decreased by 50% in the small gap, increased by 74.96% in the medium gap, and increased by 2.94% in the large gap. The content of soil-available potassium between different soil layers was significantly lower than that of the control. Soil moisture showed significant changes among different forest gaps, between different soil layers and under the interaction between them (Table 2). The soil bulk density decreased significantly compared with the control, and the surface soil reached the minimum in the medium gap.

**Table 1:** Soil physicochemical properties under different size forest gaps.

Treatment	Soil depth (cm)	pH	SOM (mg/kg)	AP (mg/kg)	AN (mg/kg)	AK (mg/kg)	SM (%)	BD (g /cm^3^ )
CK	0–10	4.07 ± 0.03a	29.70 ± 0.52b	6.88 ± 1.26a	365.71 ± 4.89b	100.58 ± 1.48a	33.44 ± 0.65b	1.87 ± 0.12a
	10–20	4.08 ± 0.06A	27.25 ± 2.71C	5.51 ± 0.48AB	318.26 ± 16.35B	96.74 ± 3.02A	32.89 ± 0.57A	1.97 ± 0.07A
SG	0–10	4.1 ± 0.05a	27.41 ± 1.27b	6.39 ± 2.62a	182.87 ± 8.05c	84.56 ± 0.96b	27.82 ± 1.02c	1.87 ± 0.12ab
	10–20	4.16 ± 0.06A	25.70 ± 1.30C	5.14 ± 2.29B	264.54 ± 38.51B	79.11 ± 0.63B	28.47 ± 1.19B	1.89 ± 0.02AB
MG	0–10	4.13 ± 0.07a	53.65 ± 6.44a	5.78 ± 2.08a	639.90 ± 29.80a	78.36 ± 2.57c	38.83 ± 3.49a	1.41 ± 0.03c
	10–20	4.13 ± 0.06A	43.34 ± 5.33B	8.07 ± 0.84A	461.62 ± 71.91A	81.84 ± 1.00B	30.01 ± 1.15B	1.45 ± 0.03C
LG	0–10	4.07 ± 0.01a	50.89 ± 5.10a	5.78 ± 1.53a	376.49 ± 53.88a	74.63 ± 0.91d	29.15 ± 1.24c	1.81 ± 0.01b
	10–20	4.14 ± 0.03A	52.06 ± 0.77A	6.79 ± 1.42AB	441.07 ± 1.20A	75.12 ± 1.34C	25.59 ± 1.14C	1.88 ± 0.02B

**Notes.**

Note: SG stands for small gaps. MG stands for medium gaps. LG stands for large gaps. Different lowercase letters mean significant differences among each forest gap of 0–10 cm soil layer at *p* < 0.05. Different capital letters mean significant differences among each forest gap of 10–20 cm soil layer at *p* < 0.05.

**Table 2:** Results of two-way ANOVA for the effects of forest gap (FG), soil layers (SL), and their interactions (FG × SL) on soil physicochemical properties. (DF: Degrees of freedom).

Index		FG	SL	FG × SL
pH	F	1.616	3.556	0.657
	P	0.225	0.078	0.591
	DF	3	1	3
SOM	F	76.177	4.953	2.698
	P	<0.001	0.041	0.081
	DF	3	1	3
AP	F	0.47	0.059	1.644
	P	0.705	0.811	0.219
	DF	3	1	3
AN	F	82.729	1.754	16.033
	P	<0.001	0.204	<0.001
	DF	3	1	3
AK	F	222.695	3.735	8.696
	P	<0.001	0.071	<0.001
	DF	3	1	3
SM	F	30.691	23.149	10.946
	P	<0.001	<0.001	<0.001
	DF	3	1	3
BD	F	91.924	1.582	0.340
	P	<0.001	0.227	0.797
	DF	3	1	3

**Notes.**

SG stands for small gaps. MG stands for medium gaps. LG stands for large gaps.

### Plant diversity and community composition

The composition of shrub layer plant forest gaps was different, and the dominant species also changed gradually (Table 3). A total of 21 shrubs appeared in the control treatment, mainly *Cinnamomum camphora* (L.) Presl*, Aralia chinensis* (L.)*, Urena lobata* (L.)*, Ardisia pusilla* A. DC. and *Cryptomeria fortunei* Hooibrenk ex Otto et Dietr. Under SG treatment, the number of plant species in the shrub layer was 28, and the dominant species gradually evolved into *Cinnamomum camphora* (L.) Presl*, Aralia chinensis* (L.)*, Urena lobata* (L.) and *Paulownia fortunei* (Seem.) Hemsl. There were 35 shrub species in the MG treatment, which were the most abundant in all treatments. *Cinnamomum camphora* (L.) Presl*, Aralia chinensis* (L.)*, Urena lobata* (L.)*, Ardisia pusilla* A. DC. and *Ardisia japonica* (Thunb) Blume were the dominant species in this treatment. The dominant species in the LG treatment were *Cinnamomum camphora* (L.) Presl, *Aralia chinensis* (L.), *Ardisia pusilla* A. DC. and *Rubus pirifolius* Smith, with a total of 29 shrub species.

**Table 3:** Main species composition of shrub layer under different forest gaps.

Serial number	Species	Important value (%)			
		Treatments			
		CK	SG	MG	LG
1	*Cinnamomum camphora* (L.) Presl	15.05	20.35	9.71	13.35
2	*Aralia chinensis* (L.)	7.02	13.86	6.20	23.27
3	*Urena lobata* (L.)	7.66	7.59	10.15	2.36
4	*Quercus serrata* Murray	3.76	3.56	4.10	1.03
5	*Rubus pirifolius* Smith	3.17	3.47	1.25	5.91
6	*Cryptomeria fortunei* Hooibrenk ex Otto et Dietr.	10.91	2.24	1.20	–
7	*Myrsine africana* (L.)	2.59	–	3.11	4.37
8	*Smilax china* (L.)	1.15	3.39	1.14	3.19
9	*Rubus lambertianus* Ser.	1.66	1.88	–	0.74
10	*Rubus chroosepalus* Focke	1.34	3.38	1.46	–
11	*Eurya japonica* Thunb.	–	2.27	2.12	1.99
12	*Ficus pandurata* Hance	–	1.66	2.18	2.10
13	*Clematis finetiana* Lévl. et Vant.	–	2.26	1.03	1.56
14	*Litsea pungens* Hemsl.	–	2.13	1.60	2.64
15	*Rubus buergeri* Miq.	–	4.11	3.09	4.62
16	*Viburnum dilatatum* Thunb.	1.42	–	–	0.95
17	*Camellia sinensis* (L.) O. Ktze.	1.83	–	–	–
18	*Ardisia pusilla* A. DC.	21.58	–	–	13. 59
19	*Mallotus barbatus* (Wall.) Muell. Arg.	4.28	–	9.33	–
20	*Eurya loquaiana* Dunn	3.79	1.13	2.39	–
21	*Xylosma racemosum* (Sieb. et Zucc.) Miq.	3.66	–	–	–
22	*Ficus stenophylla* Hemsl.	1.28	–	0.96	0.75
23	*Smilax arisanensis* Hay.	1.15	–	–	0.78
24	*Maesa perlarius* (Lour.) Merr.	1.89	–	–	–
25	*Pericampylus glaucus* (Lam.) Merr.	2.33	2.91	–	–
26	*Melastoma normale* D. Don	2.49	3.98	3.04	–
27	*Ampelopsis delavayana* Planch.	–	0.87	1.77	1.79
28	*Smilax glabra* Roxb.	–	0.7	–	–
29	*Smilax discotis* Warb.	–	2.19	–	0.88
30	*Euscaphis japonica* (Thunb.) Kanitz	–	1.06	–	2.02
31	*Smilax ferox* Wall. ex Kunth	–	1.27	–	0.78
32	*Ardisia japonica* (Thunb.) Blume	–	3.94	7.20	–
33	*Serissa japonica* (Thunb.) Thunb. Nov. Gen.	–	1.62	–	–
34	*Viburnum utile* Hemsl.	–	–	2.32	–
35	*Rhus chinensis* Mill.	–	–	0.89	0.92
36	*Rosa cymosa* Tratt.	–	–	0.79	1.21
37	*Smilax microphylla* C. H. Wright	–	–	3.57	–
38	*Rubus corchorifolius* L. f.	–	–	1.67	1.75
39	*Parthenocissus laetevirens* Rehd.	–	–	1.44	0.79
40	*Melodinus hemsleyanus* Diels	–	–	0.90	–
41	*Trachelospermum jasminoides* (Lindl.) Lem.	–	–	0.81	–
42	*Symplocos lancifolia* Sieb. et Zucc.	–	–	1.34	–
43	*Hedera nepalensis* K. Koch	–	–	1.14	–
44	*Castanea mollissima* Blume	–	–	0.91	–
45	*Ficus heteromorpha* Hemsl.	–	–	–	1.07
46	*Sabia swinhoei* Hemsl. ex Forb. et Hemsl.	–	–	–	1.68
47	*Actinidia rubricaulis* Dunn	–	–	1.52	–
48	*Oreocnide frutescens* (Thunb.) Miq.	–	–	5.95	–
49	*Vitex negundo* (L.)	–	1.17	2.77	2.44
50	*Acanthopanax trifoliatus* (L.) Merr.	–	0.74	0.96	1.47
51	*Rubus innominatus* S. Moore	–	0.72	–	–
52	*Paulownia fortunei* (Seem.) Hemsl.	–	6.23	–	–

**Notes.**

SG stands for small gaps. MG stands for medium gaps. LG stands for large gaps.

**Table 4:** Main species composition of herb layer under different forest gaps.

Serial number	Species	Important value (%)			
		Treatments			
		CK	SG	MG	LG
53	*Parathelypteris glanduligera* (Kze.) Ching	11.97	4.45	3.69	1.90
54	*Pteridium aquilinum* (L.) Kuhn	21.30	16.17	7.70	8.21
55	*Miscanthus sinensis* Anderss.	16.18	7.46	3.45	10.74
56	*Stenoloma chusanum* Ching	11.15	5.55	–	–
57	*Arachniodes simplicior* (Makino) Ohwi	12.93	–	–	11.83
58	*Setaria plicata* (Lam.) T. Cooke	26.47	20.83	12.33	15.54
59	*Blumea megacephala* (Randeria) Chang et Tseng	–	2.24	1.76	–
60	*Arthraxon hispidus* (Trin.) Makino	–	1.12	–	9.13
61	*Oplismenus compositus* (L.) Beauv.	–	2.06	9.14	1.19
62	*Eragrostis ferruginea* (Thunb.) Beauv.	–	3.05	1.06	–
63	*Oxalis corniculata* L.	–	9.08	5.38	–
64	*Arachniodes chinensis* (Rosenst.) Ching	–	4.15	3.44	4.71
65	*Dryopteris fuscipes* C. Chr.	–	3.63	3.00	30.47
66	*Synotis nagensium* (C. B. Clarke) C. Jeffreyb et Y. L. Chen	–	2.88	–	3.91
67	*Woodwardia japonica* (L. F.) Sm.	–	–	6.89	–
68	*Solanum xanthocarpum* Schrad. et Wendl.	–	2.06	–	–
69	*Centella asiatica* (L.) Urban	–	1.29	–	–
70	*Artemisia princeps* Pamp.	–	3.38	–	–
71	*Cyperus rotundus* (L.)	–	4.00	–	–
72	*Leptochloa chinensis* (L.) Nees	–	–	–	2.38
73	*Polygonum posumbu* Buch.-Ham. ex D. Don	–	–	2.16	–
74	*Lophatherum gracile* Brongn.	–	–	10.44	–
75	*Microlepia hancei* Prantl	–	–	10.13	–
76	*Dicranopteris dichotoma* (Thunb.) Bernh.	–	–	4.93	–
77	*Gonostegia hirta* (Bl.) Miq.	–	–	3.10	–
78	*Liriope spicata* (Thunb.) Lour.	–	–	1.06	–
79	*Commelina communis* (L.)	–	–	1.66	–
80	*Iris tectorum* Maxim.	–	–	2.47	–
81	*Aster tataricus* L. f.	–	–	6.92	–

There were a few species of herbaceous plants in the control treatment, most of which were pteridophytes, and there were 6 species in the herb layer (Table 4). There were 17 species of herbs in treatment SG. *Pteridium aquilinum* (L.) Kuhn and *Setaria plicata* (Lam.) T. Cooke were the dominant herbs of this treatment. The MG treatment contained the most abundant herbaceous plant species, with a total of 20 species. *Lophatherum gracile* Brongn., *Microlepia hancei* Prantl and *Setaria plicata* (Lam.) T. Cooke were the dominant species in this treatment. There were 11 species of herbaceous plants in the LG treatment, and the dominant species were *Dryopteris fuscipes* C. Chr., *Miscanthus sinensis* Anderss., *Arachniodes simplicior* (Makino) Ohwi and *Setaria plicata* (Lam.) T. Cooke.

**Figure 2:**
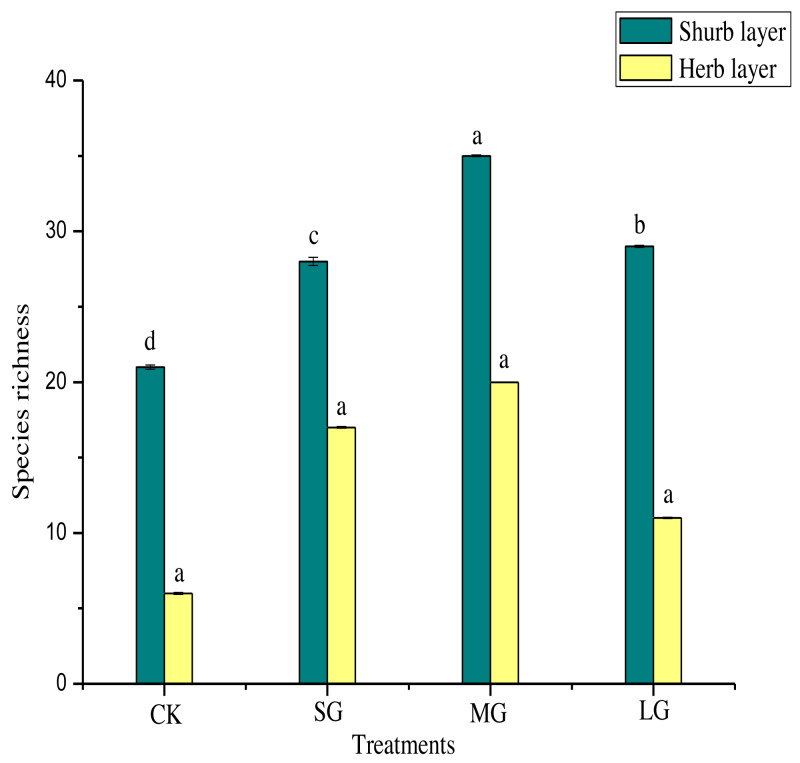
Species richness of different forest gaps. Different lowercase letters mean significant differences among each group at *p* < 0.05.

Figure 2 shows that, compared with the control, the richness index of the shrub layer in different gaps increased significantly (*P* < 0.05). The richness index of the shrub layer was the highest in the MG treatment. As shown in Table 5, the Shannon-Wiener index, Simpson index and Pielou index of the shrub layer in all treatments were significantly higher than those of the control (*P* < 0.05). The order of them was MG > SG > LG > CK. The Simpson index and Pielou index of the herb layer in different forest gaps were significantly lower than those of CK (*P* < 0.05). The Simpson index was CK > MG > SG > LG, and the Pielou index was CK > MG > LG > SG. The Shannon-Wiener index was significantly higher for CK (*P* < 0.05), but the MG treatment had the largest value.

### Relationship between soil physicochemical properties and undergrowth plant diversity

**Figure 3:**
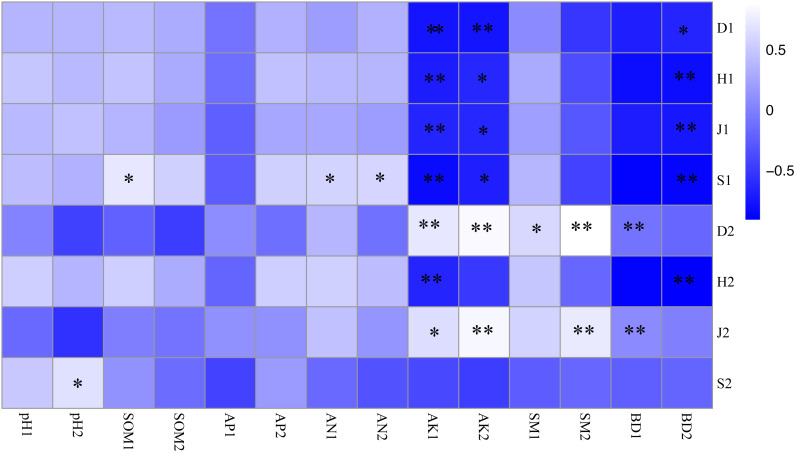
Correlation coefficient between plant diversity and soil physicochemical properties. * *P* < 0.05; ** *P* < 0.01 S1: The richness index of the shrub layer. S2: The richness index of the herb layer; H1: The Shannon-Wiener index of the shrub layer. H2: The Shannon-Wiener index of the herb layer; D1: The Simpson index of the shrub layer. D2: The Simpson index of the herb layer. J1: The Pielou index of the shrub layer. J2: The Pielou index of the herb layer. * Significant correlation. * *: Highly significant correlation. pH1, SOM1, AP1, AN1, AK1, SM1 and BD1 represent soil physicochemical properties of 0–10 cm soil layer. pH2, SOM2, AP2, AN2, AK2, SM2 and BD2 represent soil physicochemical properties of 10–20 cm soil layer.

As shown in Fig. 3, the soil-available potassium content was closely related to undergrowth plant diversity, and there was a significant negative correlation between the soil-available potassium content and the plant diversity of shrub layers (*P* < 0.01). The Simpson index of herb layers was influenced by many soil factors and was positively correlated with the available potassium, soil moisture content and bulk density of topsoil (*P* <0.05). There were significant positive correlations between the Pielou index and the available potassium content, bulk density of topsoil and soil moisture content of bottom soil (*P* <0.05). The richness index of the shrub layer showed a positive correlation not only with hydrolysis nitrogen content (*P* < 0.05) but also with soil organic matter content in topsoil (*P* < 0.01). The richness index of herb layers was significantly positively correlated with bottom soil pH (*P* < 0.05). Bottom soil bulk density of 10–20 cm was significantly negatively correlated with plant diversity (*P* < 0.05) and with herb layers (*P* <0.01).

**Table 5:** Plant diversity indexes of shrubs and herbs at different forest gaps.

Treatment	Shrub			Herb		
	Simpson	Shannon-Wienner	Pielou	Simpson	Shannon-Wienner	Pielou
CK	0.77 ± 0.01c	0.94 ± 0.01d	0.72 ± 0.01d	0.94 ± 0.01a	0.76 ± 0.01d	0.99 ± 0.01a
SG	0.93 ± 0.02a	1.27 ± 0.04b	0.87 ± 0.02b	0.86 ± 0.02c	0.98 ± 0.02b	0.79 ± 0.01d
MG	0.95 ± 0.03a	1.4 ± 0.02a	0.91 ± 0.03a	0.92 ± 0.01b	1.17 ± 0.02a	0.9 ± 0.02b
LG	0.88 ± 0.01b	1.15 ± 0.01c	0.79 ± 0.01c	0.83 ± 0.01d	0.90 ± 0.01c	0.85 ± 0.02c

**Notes.**

SG stands for small gaps. MG stands for medium gaps. LG stands for large gaps. Different lowercase letters mean significant differences among each forest gap at *p* < 0.05.

**Figure 4:**
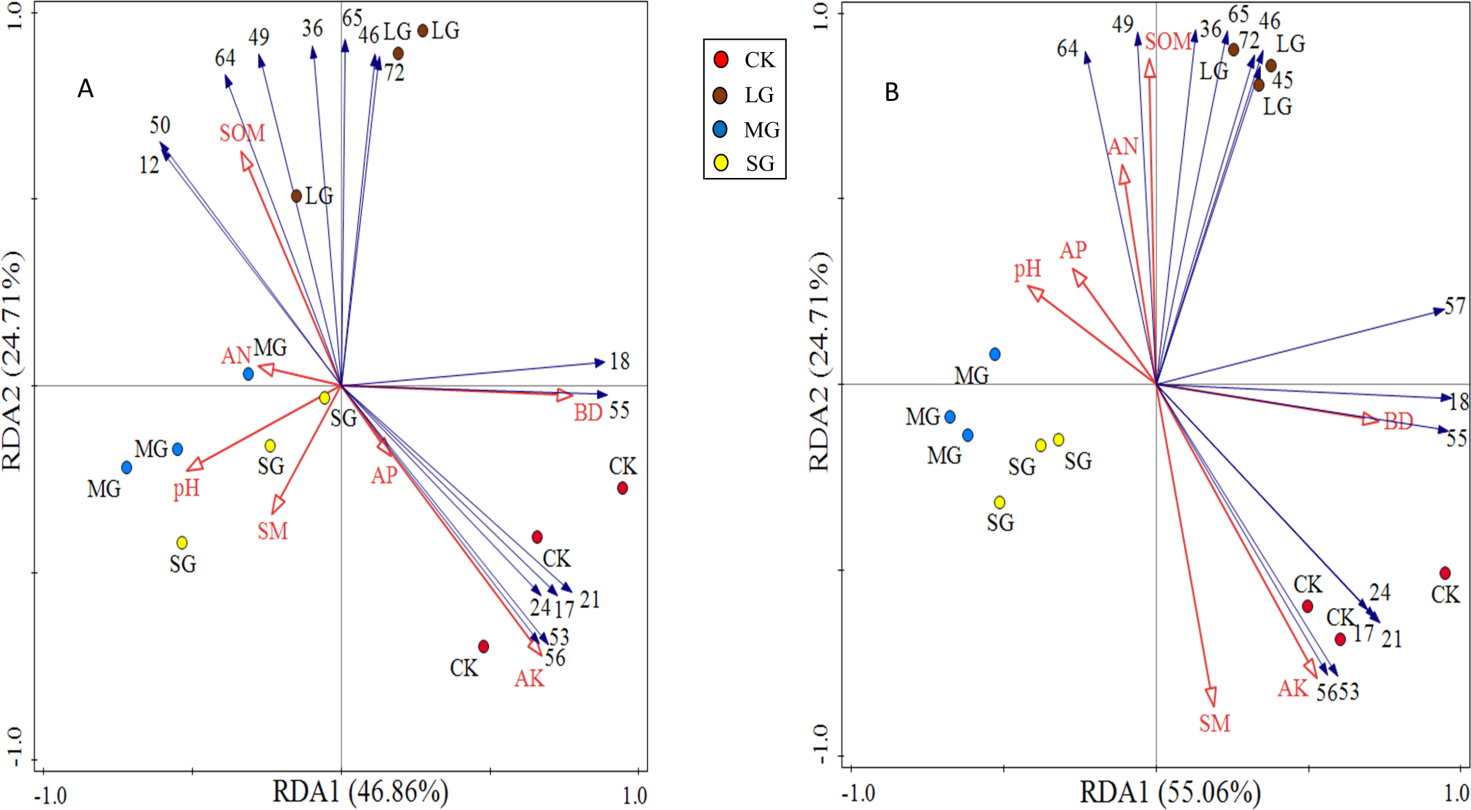
RDA analysis plot showing the relationships between soil physicochemical properties and shrub and herb composition at soil depth layers of 0–10 (A) and 10–20 cm (B), respectively. The numerical serial number represents different kinds of plants. Refer to Tables 3 and 4 for the specific meaning. The figures show only the top 15 plants in terms of relative abundance.

The redundancy analysis of the relationship between species composition in the shrub and herb layers and soil environmental factors under different forest gaps showed that the zoning of each treatment was obvious. The three forest gap treatments were visually separated from control, indicating that there were great differences in species composition and habitats (Fig. 4). Species of the shrub and herb layers are represented by blue arrow lines, and soil environmental factors are indicated by red arrow lines.

The first principal component axis and the second principal component axis explained 46.86% and 24.71% of the species composition of the 0–10 cm soil layer, respectively, and the total explanation rate reached 71.57% (Fig. 4A). The first principal component axis and the second principal component axis explained 55.06% and 26.54% of the species composition of the 10–20 cm soil layer, respectively, and the total explanation rate reached 81.60% (Fig. 4B). All these indicated that soil physicochemical properties could explain the differentiation of the spatial and characteristics of the vegetation community under the forest. Soil available potassium, bulk density and soil organic matter made a great contribution to the species composition in shrub and herb layers, which were the main factors significantly affected the distribution of species composition (Table 6).

**Table 6:** Soil physicochemical properties explanatory variables and their contributions to the vegetation composition.

Index	Soil depth (cm)	Explains %	Contribution %	F	P
AK	0–10	34.4	39.9	5.2	0.008^**^
	10–20	28.5	29.5	31	0.002^**^
BD	0–10	23.1	26.8	4.9	0.036^*^
	10–20	36	37.3	5.6	0.004^**^
SOM	0–10	23	26.7	9.5	0.014^*^
	10–20	28.2	29.2	7.1	0.006^**^
AN	0–10	3	3.4	1.3	0.302
	10–20	0.9	0.9	1.2	0.324
AP	0–10	0.9	1	0.3	0.63
	10–20	0.8	0.9	1	0.376
SM	0–10	0.9	1	0.3	0.692
	10–20	0.3	0.3	0.3	0.772
pH	0–10	1.1	1.2	0.3	0.608
	10–20	1.7	1.8	2.2	0.126

**Notes.**

** *P* < 0.01.

* *P* < 0.05.

## Discussion

### Responses of soil physical and chemical properties to different gap sizes

Except for the significant increase in soil moisture in the middle gaps, the soil physical properties in the gaps were significantly lower than those in the control, which was basically consistent with our previous hypothesis. Soil moisture influences soluble nutrient uptake and microbial activity, which are determined by and closely related to plant growth (Gao et al., 2020). The increase of soil moisture in the middle gaps may be due to the close relationship between soil moisture and plant growth. However, the plant composition in this kind of forest gap is the most, so the soil moisture is significantly higher than that of other treatments. Studies have shown that soil moisture in different sizes of forest gaps will show a complex pattern, and there is no obvious law (Pang et al., 2016). Our study showed that only the soil moisture of the medium gap was significantly higher than that of the control, but the soil moisture of the large forest gap and small forest gap was significantly lower than that of the control (Table 1). For small canopy gaps, the gap formed by artificial felling will destroy the plants under the forest when the felled *P. massoniana* is removed so that the interception function of the plants in the forest is lower than that of the control and the soil moisture in the gaps decreases (Meyer, Sisk & Wallace Covington, 2001; Simonin et al., 2007). For larger canopy gaps, in addition to more rainfall in the forest, the increased soil water evaporation and solar radiation in the forest gap will also have a negative impact on soil moisture (He et al., 2013; Ma et al., 2014; Han et al., 2020). These contradictory processes are likely to complicate the relationship between soil moisture and gap size. Soil bulk density is crucial in soil development (Mora & Lázaro, 2014). Soil bulk density can not only affect water infiltration and root growth but also play an important role in the maintenance of water capacity and plant nutrient availability (Salazar et al., 2009). In this study, soil bulk density showed a parabola with the change in gap size, reached the lowest point in the medium gap, and then gradually increased, and bulk density typically increased as soil depth increased. The setting of the forest gap improves the compactness of soil.

Soil organic matter and hydrolyzed nitrogen stocks are controlled by the complex interplay of soil physical, chemical, and biological conditions (Li & Nie, 2020a). In this study, compared with other larger forest gaps, soil organic matter and hydrolyzed nitrogen were significantly higher than those of the control, and the opposite trend of soil organic matter and hydrolyzed nitrogen appeared in the small forest gap (Table 1). With the increase in gap area, the content of soil available potassium decreased significantly, which may be because in the *P. massoniana* plantation, the fallen needles may stay on the forest ground for a long time, resulting in slow decomposition, which may eventually reduce the nutrient absorption of trees. At the same time, withered pine needles can also prevent other plant litter from decomposing and being absorbed by the soil (Chen & Cao, 2014; Ali et al., 2019). In summary, the medium gap can absorb soil water very well, improve the soil bulk density to a great extent and is conducive to the accumulation of soil organic matter and hydrolyzed nitrogen, which plays a positive role in the environment of the plantation. However, the conclusions of this study can only be applied to similar ecological environment. Therefore, the responses of plant diversity and soil physicochemical properties to other environmental conditions are recommended to be studied in the future.

### Responses of undergrowth plant diversity to different gap sizes

The gap size is the direct “driving force” for natural regeneration in forest gaps (Zhang et al., 2018). The species composition of shrubs and herbs in the forest gap changed obviously, and they were in the stage of competition and coexistence among populations. Sufficient light and rainwater led to the formation of plant communities under forest gaps of different sizes (Jokela, Siitonen & Koivula, 2019). The initial period of the formation of the forest gap increased the light intensity in the forest, promoted the seed germination of solar plants in the woodland, provided suitable environmental conditions for the settlement of plant seedlings, and reduced the proportion of shade-tolerant plants. However, with the continuous development of the gap, the light transmittance is gradually weakened and the distribution is patchy, which leads to the emergence of different shade-tolerant tree species, making the species composition in the gap more abundant. There were the most abundant plant species in the medium gaps (Fig. 2), and the number of light-loving plants under the shrub layer increased greatly (Table 3). The proportion of shade-tolerant plants in herb layer also increased significantly, and there were many shade-tolerant species that did not appear in other treatments, such as *Lophatherum gracile* Brongn, *Micronesia hancei* Prant, *Gonostegia hirta* (Bl.) Miq, *Liriope spicata* (Thunb.) Lour., and *Aster tataricus* L. f. (Table 4). Neutral plants also account for a part of medium gaps. But in the large gaps, the proportion of light-loving plants increased the most, and the important value of *Aralia chinensis* (L.) was much higher than that of other treatments, and the proportion of *Myrsine africana* (L.), *Acanthopanax trifoliatus* (L.) Merr., *Euscaphis japonica* (Thunb.) Kanitz, *Rosa cymosa* Tratt. and *Rubus corchorifolius* L. f. should not be underestimated. Due to the heterogeneity of the gap environment, different species have different responses to the gap, which makes the diversity of shrubs and herbs different in the gap.

Gaps in forest canopies play a major role in local species richness because they increase habitat heterogeneity and accelerate the development of more complex forests (Swartz et al., 2020; Tena et al., 2020). In this study, the significant increase in species richness of the shrub layer in the forest gap may be because sufficient sunlight in the gap can cause more plants to grow under the forest (Wang et al., 2019b). The maximum value of species richness of the shrub layer appeared in the medium gap (Fig. 2). Generally, the herb layer has stronger sensitivity than the shrub layer. However, there was no practical relationship between the species richness index and forest gap sizes in this study, contrary to the previous hypothesis, which may be due to the weak sensitivity of herbaceous plants to the light response in forest gaps (Nowińska, 2010). The Shannon-Wiener index, Simpson index and Pielou index of the shrub layer and herb layer in the forest gap showed the opposite trend. The Shannon-Wiener index, Simpson index and Pielou index of the herb layer were significantly lower than those of the control (Table 5). This result shows that the herb layer is vulnerable to external interference, and the sharp changes in plant species and quantity make the distribution of different species in the biological community more uneven, and the ecological function of herbaceous plants is not prominent (Wang & Zhang, 2019a). The light transmittance in the large area gap is higher than that in the medium or small area gap, so the light environment in the large area gap is too bare and the dry and wet distribution in the gap is uneven such that the plant growth is relatively slow, which is more suitable for the growth of solar plants (Ritter, Dalsgaard & Einhorn, 2005). However, in the medium gap, the environment provided for plant growth is conducive not only to the growth of light-loving plants but also to the growth of shade-tolerant or neutral plants in the process of increasing light. Therefore, the medium gap can maximize the plant diversity under the forest. In a biological system of enormous heterogeneity and complexity, the same forest management measures may also draw different conclusions, so the popularization of our conclusions will also be limited.

### Interaction between undergrowth plant diversity and soil physical and chemical properties

It is well known that species composition cause changes in soil properties that lead to complex local interactions between vegetation and soil (Pérez-Bejarano et al., 2010). In the soil-vegetation ecosystem, soil and vegetation interact with each other (Li, Wang & Zhang, 2020c). Different forest ecosystems possess different plant-soil interactions. In our study, the species diversity in the forest gap was closely related to the soil available potassium content, surface soil bulk density and soil moisture (Fig. 3). There was considerable correlation between the content of available potassium in soil and the parent material of the soil itself. Current reports show that potassium limits plant productivity in terrestrial ecosystems due to limited nitrogen and phosphorus at a global level (Sardans & Peñuelas, 2015; Li et al., 2021). In this study, the content of soil-available potassium was negatively correlated with the plant diversity index of the shrub layer but positively correlated with the Simpson index and Pielou index of the herb layer, indicating that available potassium was the limiting factor of the shrub layer. The content of soil-available potassium was easy to eluviate due to rain water erosion in the early stage of the forest gap, and the plant growth of the shrub layer produced negative feedback (Cox et al., 1999). Herbaceous plants have low requirements for soil nutrients, so they can continue to grow even under the loss of potassium ions (Wang et al., 2018). Soil moisture content and soil bulk density are important for soil hydrological processes (Grote et al., 2010). Improvement of these two physical properties plays an important role in guiding forestry management and improving forest productivity (Boluwade & Madramootoo, 2016). Soil moisture is the main factor affecting direct or indirect changes such as rainwater, soil erosion, runoff and plant growth (Wang et al., 2012; Sun et al., 2020). Forest gaps formed by felling can affect the infiltration of the soil moisture layer and deep layer and affect the participation of underground plants and microorganisms in the geochemical cycle (Vereecken et al., 2014). Therefore, the soil moisture content and soil bulk density were closely related to the plant diversity of the shrub layer and herb layer. Soil organic matter is vital to biodiversity to maintain many functions of plant growth (Kooch & Bayranvand, 2019; Kooch, Ehsani & Akbarinia, 2020). Increasing the litter amount in the forest gap accelerated soil microbial decomposition and promoted an increase in the organic matter content in topsoil (Bongiorno et al., 2019). The soil organic matter content increased significantly with shrub layer species richness and affected the degree of increase of the shrub layer species to some extent. Moreover, the soil bulk density, soil available potassium and soil organic matter content also significantly influenced the vegetation distribution in the shrub and herb layers (Fig. 4) (Table 6). Compared with the control treatment, soil physicochemical properties and plant distribution were significantly improved under different forest gap sizes.

## Conclusion

When forest management is carried out, attention should be paid to promoting the natural regeneration of forests and achieving sustainable development. The size setting of forest gaps is very important for accomplishing these goals. Our results indicated that there were obvious differences among vegetation communities under different forest gap sizes. The forest gap of the shrub layer was significantly improved over that of the herb layer and had a significant effect on the soil physical and chemical properties. Soil-available potassium was the main limiting factor for the growth of undergrowth plants. Soil bulk density and soil organic matter content also significantly affected vegetation distribution. Under the medium forest gap, not only were the soil physical and chemical properties promoted most notably, but plant diversity and distribution of the shrub layer were also prominent. If we strictly focus on improving woodland quality and increasing plant diversity, our research results recommend setting a medium gap for the near-natural development of forest plantations. At the same time, we also need to further extend the time limit to quantify the differences in soil properties between different gaps in different plantation ecosystems and consider the plant distribution under the gaps in order to accurately determine which forest gap size can have the most significant effect on the improvement of plantation quality.

##  Supplemental Information

10.7717/peerj.12222/supp-1 Supplemental Information 1 Plant diversity, plant composition and soil physical and chemical properties

## Additional Information and Declarations

### Data Availability

The following information was supplied regarding data availability: The raw measurements are available in the Supplementary File.
